# Impact of SGLT-2 Inhibitors on Biomarkers of Heart Failure

**DOI:** 10.3390/cells14120919

**Published:** 2025-06-18

**Authors:** Dana Darwish, Pooja Kumar, Khushi Urs, Siddharth Dave

**Affiliations:** 1 Department of Anesthesiology and Pain Medicine, University of Texas Southwestern, Dallas, TX 75390, USA; 2 School of Medicine, University of Texas Southwestern, Dallas, TX 75390, USA; 3 University of Texas Southwestern Medical Center, Dallas, TX 75390, USA

**Keywords:** SGLT-2 inhibitors, heart failure, diabetes mellitus, cardiac biomarkers

## Abstract

Type 2 diabetes mellitus affects nearly 7% of the world’s population and is a significant contributor to the development of cardiovascular disease and heart failure. Historically, the pharmacologic therapy of cardiovascular disease has centered around blood pressure control, insulin and cholesterol management, the inhibition of the renin–angiotensin system, and catecholamine blockade. Recent evidence suggests that sodium–glucose cotransporter 2 (SGLT-2) inhibitors provide significant cardiovascular protection to patients with and without diabetes. The use of SGLT-2 inhibitors is associated with significant changes to serum biomarkers of cardiac function. In this narrative review, we summarize how biomarkers reflect physiologic aspects of cardiovascular function and how these are affected by the use of SGLT-2 inhibitors.

## 1. Introduction

Cardiovascular disease (CVD) is a leading cause of morbidity and mortality across the globe [1]. In the United States, approximately 10% of adults over the age of 20 are affected by CVD, equivalent to 28.6 million individuals nationwide [2]. It is one of the primary causes of death in the U.S., significantly reducing quality of life and placing a substantial economic burden on individuals and the healthcare system. While advancements in the medical management of CVD have reduced age-standardized mortality rates, the overall CVD burden is still projected to increase due to a global aging population [3]. Between 2025 and 2050, the prevalence of CVD is expected to increase by as much as 90% [4].

Type II diabetes mellitus (T2DM) further compounds this growing burden. Globally, an estimated 537 million adults between the ages of 20 and 79 have T2DM [5]. T2DM is a well-established risk factor for CVD, significantly increasing the likelihood of adverse cardiovascular events. Among individuals with T2DM, approximately 33% have coexisting CVD, and over 70% ultimately die of CVD-related complications [6,7]. Historically, the mainstay of the pharmacologic treatment of CVD has been blood pressure, insulin, and cholesterol control; the inhibition of the renin–angiotensin–aldosterone (RAAS) system; and catecholamine blockade. This is in addition to lifestyle modification, which is also an important management strategy for T2DM. However, the introduction of sodium–glucose cotransporter 2 (SGLT-2) inhibitors has transformed the landscape of T2DM and CVD treatment, offering cardiovascular benefits that extend beyond glycemic control [7,8].

Four SGLT-2 inhibitors are approved for use in adults: canagliflozin, dapagliflozin, empagliflozin, and ertugliflozin [9]. An emerging area of interest is the effect of SGLT-2 inhibitors on cardiac biomarkers. Given that SGLT-2 inhibitors do not have direct receptors on the heart, their observed cardioprotective effects are likely mediated through indirect mechanisms involving the modulation of interstitial edema, insulin resistance, energy metabolism, remodeling, and inflammation [10]. In this narrative review, we will explore the physiology of key biomarkers of CVD and examine the relationship between SGLT-2 inhibitor use and expression of these biomarkers.

## 2. Cardiovascular Disease

CVD encompasses a broad spectrum of pathologies, including congenital disease, peripheral vascular disease, aortic disease, coronary artery disease, heart failure, and cardiac arrhythmias. Among these, ischemic heart disease is one of the most common causes of morbidity and mortality. In 2019, the prevalence of ischemic heart disease was estimated to be 197 million of the total 523 million cases of CVD worldwide [3].

Cardiomyopathies, a major subset of CVD, are broadly categorized into ischemic and non-ischemic types. Ischemic cardiomyopathy typically results from coronary artery disease, leading to myocardial ischemia and acute coronary syndromes, such as angina and myocardial infarction. Ischemic cardiomyopathy is the leading cause of heart failure in developed countries [11]. In contrast, non-ischemic cardiomyopathy may be triggered by a diverse range of etiologies, including genetics, infiltrative disorders (e.g., amyloidosis, sarcoidosis), infections, autoimmune conditions, medication-induced toxicity, hypertensive urgency/emergency, peripartum cardiomyopathy, alcoholic/cirrhotic cardiomyopathy, and idiopathic. Non-ischemic cardiomyopathy is further classified into dilated cardiomyopathy, hypertrophic cardiomyopathy, and restrictive cardiomyopathy [12,13]. Among these, dilated cardiomyopathy is the most common cause of heart failure, accounting for approximately 30 to 40% of patients in multicenter heart failure trials [14].

Heart failure (HF) is characterized by structural or functional cardiac abnormalities that lead to poor ventricular filling or ejection with resultant edema or vascular congestion [15]. As of 2024, an estimated 6.5 million Americans and 64 million individuals worldwide had been diagnosed with HF [16]. HF is typically classified by left ventricular ejection fraction into three subtypes: HF with reduced ejection fraction (HFrEF; ejection fraction ≤ 40%,), HF with mildly reduced ejection fraction (HFmrEF; ejection fraction 41–49%), and HF with preserved ejection fraction (HFpEF; ejection fraction ≥ 50%) [17]. HFrEF represents approximately 50% of all heart failure cases, while HFpF and HFmrEF account for 30% and 20%, respectively [18,19]. In the United States, an estimated 45% of patients hospitalized for HF exacerbations present with HFrEF [18].

## 3. Diabetes Mellitus

T2DM is a well-established risk factor for HF and CVD. As of 2021, over 29 million American adults and 537 million individuals worldwide were reported to be living with T2DM, with the global prevalence rising by 30% in the last decade [16,20]. The presence of T2DM in patients with HF is associated with increased all-cause mortality. In the CHARM trial, patients with T2DM faced a significantly higher risk of death from any cause, with the risk increasing by approximately 60% in both HFpEF (HR 1.59) and HFrEF (HR 1.6) [21]. T2DM also increases the risk of cardiovascular death and hospitalization. According to the I-PRESERVE trial, cardiovascular death or HF hospitalization occurred in 34% of patients with T2DM and HFpEF versus 22% in those without T2DM [16]. The total annual cost of diabetes care in the United States, as of 2022, was estimated at USD 306 billion, with individuals with T2DM accounting for a quarter of all healthcare dollars spent [22]. Given the increasing prevalence of T2DM and its compounding effect on HF outcomes, a deeper understanding of the interplay between diabetes and HF is of growing importance.

**Figure 1:**
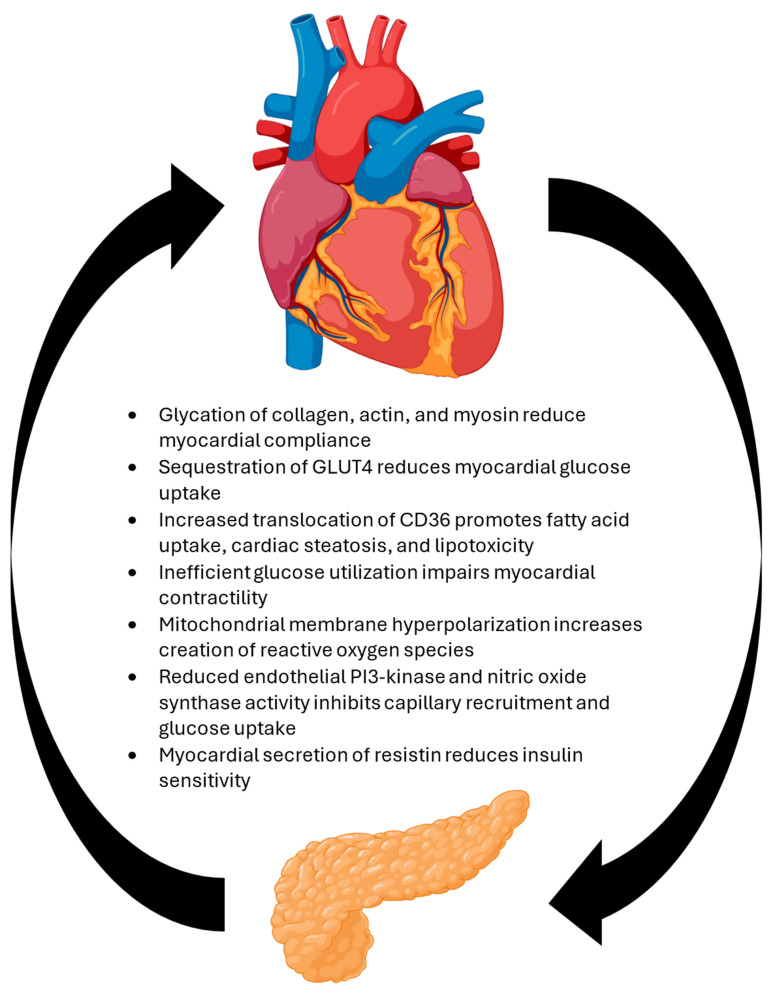
Multiple mechanisms contribute to the relationship between diabetes and heart failure, including abnormal glucose handling, inefficient energy metabolism, lipotoxicity, and microvascular dysfunction.

HF and T2DM frequently coexist and demonstrate a bidirectional relationship: the presence of one increases the risk of developing the other. (Figure 1) Multiple mechanisms contribute to this. Hyperglycemia promotes the glycation of collagen matrix proteins, actin, and myosin, resulting in increased myocardial stiffness [23]. Insulin resistance promotes the internalization of the GLUT4 glucose transporter, reducing myocardial glucose availability and increasing fatty acid utilization [24]. This, along with the hyperpolarization of the mitochondrial membrane through increased nicotinamide adenine dinucleotide and flavine adenine dinucleotide flux, reduces the efficiency of energy production and increases reactive oxygen species (ROS) production [25]. Increased fatty acid availability stimulates the translocation of the CD36 fatty acid transporter to the sarcolemma, promoting further fatty acid accumulation, cardiac steatosis, and lipotoxicity [26]. Dysregulated glucose handling and symptomatic HF are also associated with the increased secretion of resistin, further impairing insulin resistance [27]. Reduced cardiac output may also reduce insulin-mediated glucose uptake by reducing endothelial PI3-kinase and nitric oxide synthase-dependent capillary recruitment [28].

When both HF and T2DM are present, there is a marked increase in mortality, hospitalization, reduced quality of life, and heightened economic burden. In the multinational CAPTURE study, the prevalence of T2DM among patients with HF ranged from 4.3 to 28%, while among patients with T2DM, the prevalence of HF ranged from 12 to 57% [29]. In patients hospitalized with HF, the prevalence of T2DM may exceed 40% [16]. After adjusting for risk factors, large cohort studies, including the NHANES I and the Framingham Heart Study, demonstrate a 2–4-times increased risk of CVD in individuals with T2DM compared to those without [16,29,30]. T2DM is highly prevalent among patients with ischemic heart disease, with prevalence rates that range from 40 to 60% [31]. In the context of non-ischemic cardiomyopathy, dilated cardiomyopathy has the highest demonstrated mortality rate, with a 15.2% mortality in patients with coexisting T2DM [32]. Although few studies have directly compared the prevalence of T2DM in HFrEF versus HFpEF, existing data suggest similar rates of T2DM between subtypes. Among patients hospitalized for HF, T2DM was found in approximately 40% of individuals in each cohort, affecting HFpEF and HFrEF groups at comparable rates [30].

## 4. Direct Mechanism of Action of SGLT-2 Inhibitors

**Figure 2:**
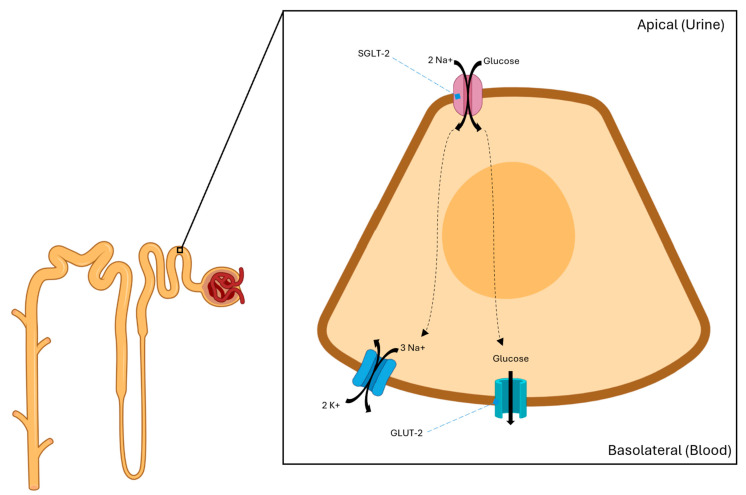
Filtered glucose is reabsorbed from the proximal convoluted tubule via SGLT-2 on the apical surface of the tubular epithelium, then transported to the serum via GLUT-2 at the basolateral surface. Inhibition of SGLT-2 results in glucosuria and natriuresis.

SGLT-2 is a transmembrane helical protein expressed in the proximal convoluted tubules of the kidneys and exerts its physiological function by reabsorbing filtered glucose and sodium, thus enhancing glucose and sodium retention [33,34]. (Figure 2).

SGLT-2 inhibitors are synthetic analogs of the naturally occurring O-glycoside phlorizine that prevent the reabsorption of filtered glucose and sodium, promoting urinary glucose excretion and natriuresis [35]. By increasing distal solute delivery, SGLT-2 inhibitors also increase sodium sensing at the macula densa, thus activating tubuloglomerular feedback and reducing intraglomerular pressure [36]. This serves as an important control on the progression of renal disease in T2DM, wherein chronic hyperglycemia results in increased SGLT-2 expression in the proximal convoluted tubule with enhanced glucose and sodium reabsorption and the blunting of tubuloglomerular feedback. SGLT-2 inhibitors also reduce GLUT2 protein expression in the kidneys, resulting in reduced glucose transport to the serum at the basolateral membrane [37]. SGLT-2 inhibitors induce starvation-like responses in the proximal tubules of rodents, including sirtuin/5′ AMP-activated protein kinase activation and the inhibition of the protein kinase B/mTORC1 pathway [38,39]. This alters the pathophysiology of T2DM, inducing protective autophagy and preventing inflammation and fibrosis, which leads to cellular aging and death [34]. In diabetic patients with albuminuria, SGLT-2 inhibitors increase urine metabolites linked to mitochondrial metabolism, potentially indicating improved mitochondrial function and activity [40]. The single-cell RNA sequencing of proximal tubules in db/db mice also showed that SGLT-2 inhibitors restored mitochondrial function and fatty acid oxidation in the proximal tubules [41]. In nondiabetic mice, SGLT-2 inhibitors altered kidney metabolism, resulting in upregulated renal gluconeogenesis [42,43,44].

Another benefit of SGLT-2 inhibitors is their ability to reconfigure metabolite transporters in the early proximal tubules of the kidneys. Data shows evidence of physical and functional coupling of SGLT-2 with sodium and metabolite transporters in tubular luminal membranes. This inhibitory interaction of SGLT-2 inhibitors is thought to involve scaffolding proteins like MAP17 but can also relate to changes in transporter phosphorylation and the insulin-lowering effects of SGLT-2 inhibitors [34]. For example, insulin co-stimulates SGLT-2, NHE3, and the urate transporter URAT1 in the early proximal tubule, which correlates with the post-prandial rise in GFR, enhancing the amounts of filtered sodium, glucose, bicarbonate, urate, and other substrates that are reabsorbed in the early proximal tubule [34]. When hyperinsulinemia is present in states like obesity and T2DM, co-stimulation can facilitate sodium chloride, renal glucose, and urate retention, affecting a range of transport processes [34]. Several studies show either direct or indirect physical interactions of SGLT-2 with multiple other apical metabolite transport proteins in the early proximal tubule, many of which are Na-coupled, including possible interactions with early proximal tubule amino acid transport [37]. SGLT-2 inhibitors can also lower renal concentrations of uremic toxins by lowering the activity of basolateral organic anion transporters in the proximal tubule, resulting in less toxin exposure to the kidneys [45,46,47].

SGLT-2 inhibitors were originally developed to treat hyperglycemia in patients with T2DM [48]. However, given the relationship between T2DM and HF, current clinical guidelines now emphasize integrated treatment strategies targeting both conditions [49]. Cardiovascular benefits were first noted in large outcome trials designed to evaluate the safety of these agents in patients with T2DM. The landmark EMPA-REG OUTCOME trial demonstrated significant reductions in cardiovascular mortality and heart failure hospitalizations with the use of empagliflozin in patients with T2DM and established CVD [50]. It also identified renoprotective effects by slowing the decline in kidney function [51,52]. These findings were later supported by other major trials, including CANVAS and DECLARE-TIMI 58, which confirmed reductions in major adverse cardiovascular events and HF hospitalizations [53]. More recently, the DELIVER and EMPEROR-Preserved trials extended these benefits to patients with HFpEF, again demonstrating a reduction in the same composite endpoints [54]. The benefits were observed regardless of diabetes status, prompting the inclusion of SGLT-2 inhibitors in the American College of Cardiology and American Heart Association (AHA) treatment guidelines for HFrEF [17]. As a result, SGLT-2 inhibitors have transitioned from being purely glycemic control medications to cornerstone therapies in the management of HF.

## 5. Indirect Effects on Cardiovascular Physiology

### 5.1. Reduction in Edema

The DAPA-HF trial and EMPEROR-Reduced trial both showed significant reductions in the composite outcome of cardiovascular death or HF hospitalization in patients with HFrEF. The mechanisms behind these benefits are a matter of some debate but are likely related to improved electrolyte balance, renal perfusion, and interstitial edema [55]. SGLT-2 inhibitors undoubtedly provide a diuretic effect, and this reduction in fluid volume is independent of kidney function or diabetes status [56]. Traditional loop and thiazide diuretics do not demonstrate the same reduction in HF-related hospitalization; thus, these protective effects are unlikely to be the result of simple diuresis [57]. Mechanistically, this may be achieved via the modulation of non-osmotic sodium, something that is not achieved via traditional diuretics due to RAAS-mediated sodium retention [58]. In the EMPIRE HF RENAL trial, treatment with empagliflozin resulted in significant reductions in extracellular volume and plasma volume [59], while a post hoc analysis of EMPA-REG OUTCOME showed a hemoconcentration effect to have contributed significantly to the reduction in cardiovascular mortality [60].

### 5.2. Ventricular Remodeling

SGLT-2 inhibitors have been shown to reverse ventricular remodeling in patients with HF [61]. This is achieved by decreasing the stiffness of the myofilaments reducing cardiomyocyte hypertrophy and alleviating myocardial fibrosis [62,63,64]. It can also suppress the sympathetic nervous system, elicit anti-inflammatory responses, decrease oxidative stress, and reduce myocardial infarction [56,65]. For example, empagliflozin use reduces left ventricular volumes in patients with T2DM and heart failure. This leads to ventricular unloading, which ultimately reverses ventricular remodeling [66]. SGLT-2 inhibitors have anti-apoptotic effects on the heart as well, via the suppression of tumor necrosis factor alpha overexpression. Tumor necrosis factor alpha (TNF-α) overexpression induces apoptosis and leads to progressive ventricular dilatation and systolic dysfunction [67]. Empagliflozin use reduces the expression of tumor necrosis factor alpha and the production of ROS, which inhibits myocardial apoptosis [68].

### 5.3. Cardiac Energy Metabolism

In a normal heart, glucose and fatty acid oxidation contribute to approximately 90% of adenosine triphosphate (ATP) production [41]. Normally, fatty acids are the predominant fuel for energy metabolism in the heart, followed by glucose as a preferential alternative under certain pathological conditions [69,70]. However, when fatty acids and glucose oxidation are impaired, such as in heart failure, ketone bodies or branched-chain amino acids can become an alternative source of energy [71]. Ketones can generate more free energy per mole of oxygen to fuel ATP production and produce less ROS than glucose and fatty acids [72,73].

Treatment with SGLT-2 inhibitors improves the utilization of ketone bodies in HF through multiple pathways, such as increased hepatic production of ketone bodies, regulating mitochondrial metabolism, activating nutrient deprivation signaling pathway, and promoting the utilization of cardiac ketone bodies [64,74,75,76,77,78]. In addition, SGLT-2 inhibitors can improve cellular hypoxia, reduce inflammation, and allow myofibroblasts to revert to erythropoietin-producing fibroblasts, providing more oxygen for energy metabolism in the myocardium [79,80,81].

### 5.4. Inflammation

Inflammation is recognized as an important contributor to the development of HF via mechanisms, including the development of anti-cardiac antibodies, coronary endothelial dysfunction, the hypophosphorylation of titin, and an increase in cardiac myocyte stiffness and tension [82]. Danger-associated molecular patterns have been shown to modulate fibrotic, hypertrophic, and apoptotic processes in the myocardium [83]. The activation of the NLRP3 inflammasome via such mechanisms worsens the progression of HF [84]. SGLT-2 inhibitors may reduce inflammation-mediated HF progression via several mechanisms. β-hydroxybutarate is a potent inhibitor of NLRP3 activity [85]. Increased ketone utilization in the face of reduced serum glucose may reduce NLRP3 activity. SGLT-2 inhibitors may also reduce macrophage activity, as glucose is their preferred energy source [86].

### 5.5. Sympathetic Inhibition

Increased sympathetic nervous system activity is a compensatory response in the acute phase of HF [87]. Chronically, it becomes maladaptive and promotes hypertrophy and dysfunction. Chronic HF is marked by significantly elevated levels of circulating catecholamines and catecholamine spillover from sympathetic nerve terminals [88]. SGLT-2 inhibitor-mediated sympathetic nervous system inhibition has been demonstrated in both animal studies and clinical trials. Herat et al. showed reduced levels of tyrosine hydroxylase and norepinephrine in mice treated with dapagliflozin [89]. Additionally, Jordan et al. showed reduced sympathetic nerve activity in patients treated with empagliflozin compared to other diabetic medications [90].

### 5.6. Improved Peripheral Vascular Function

Arterial aging and stiffness are important predictors of worse outcomes in HF [91]. Increases in arterial stiffness and pulse pressure have been correlated with increased risk of myocardial infarction, HF, and arrhythmia [92,93,94]. SGLT-2 inhibitors may reduce arterial stiffness via several mechanisms. Dapagliflozin has been shown to attenuate endothelial cell activation and cause direct vasorelaxation [95]. Canagliflozin reduces endothelial cell activation via both AMPK-dependent and -independent pathways [96]. Empagliflozin treatment increases circulating pro-angiogenic CD133+ cells and may improve cardiovascular health by increasing the counts of cells involved in blood vessel repair [97].

### 5.7. Reduced Albuminuria

Albuminuria is an independent risk factor for the development and progression of symptoms in HF [98]. The presence of albuminuria likely reflects hemodynamic and structural changes at the glomerular level. An analysis of the TOPCAT study showed both micro- and macroalbuminuria to be associated with the development of HF [99]. Similarly, Shuvy et al. showed both micro- and macroalbuminuria to be predictive of mortality in HF [100]. Multiple studies have demonstrated a reduction in albuminuria following SGLT-2 inhibitor therapy [101,102]. In diabetic kidney disease, podocyte foot processes undergo significant remodeling, resulting in the loss of filtration barrier function, a process that is accompanied by an increase in expression of SGLT-2 [103]. Both empagliflozin and dapagliflozin have been shown to reduce SGLT-2 expression in podocytes in diabetic kidney disease and have even reduced podocyte injury [104]. Underlying mechanisms may involve direct actions on podocytes via SGLT-2 receptors and reduced podocyte damage by improving hyperglycemia [105].

## 6. Cardiac Biomarkers

Cardiac biomarkers serve as indispensable tools in the diagnosis, risk stratification, and management of various cardiovascular diseases, particularly in the context of myocardial injury and HF. According to the AHA guidelines, cardiac troponins and natriuretic peptides represent the primary biomarkers utilized in clinical practice for acute coronary syndromes and heart failure, respectively [106].

Several mechanisms have been proposed for the interactions between SGLT-2 inhibitors and cardiac biomarkers, and the cardiovascular benefits seen with the use of SGLT-2 inhibitors. In addition to inducing systemic cardiovascular changes, SGLT-2 inhibitors have demonstrated specific binding to SGLT-1, which is found in renal, intestinal, and myocardial tissues [107]. This binding is likely promoted via allosteric shape changes following the binding of Na ions to the Na2 site along the helical break of the TM1 segment of SGLT-1 [108].

**Figure 3:**
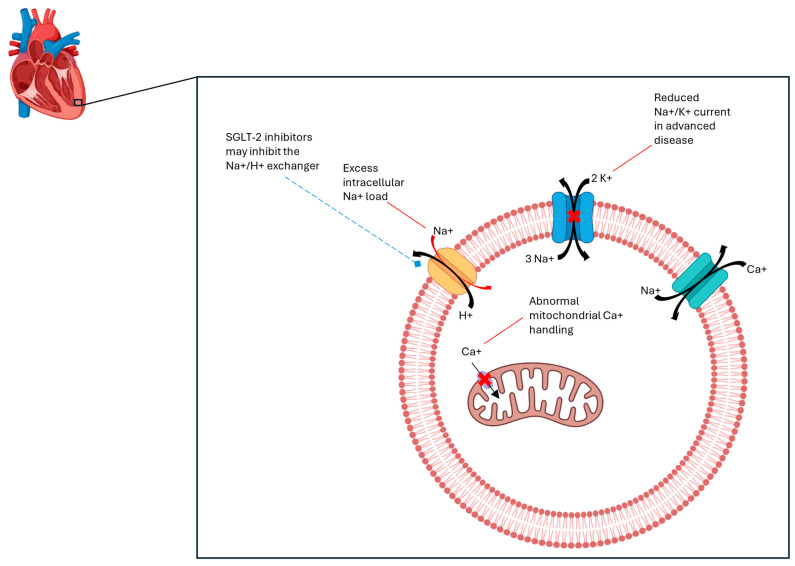
Heart failure is marked by excess intracellular Na^+^ buildup, reduced Na^+^/K^+^ activity, and impaired Ca^+^ handling within the mitochondria. Dysfunctional Ca^+^ transport across the mitochondria results in reduced myocyte energy production. SGLT-2 inhibitors reduce Na^+^/H^+^ antiporter activity, thus reducing intracellular Na^+^ and improving Ca^+^ handling and energy production.

In the early stages of heart failure, cardiac myocyte intracellular sodium concentration ([Na^+^]i) increases due to increased diastolic Na^+^ influx, with preserved Na^+^/K^+^- ATPase function [109]. (Figure 3) However, in the later stages of heart failure, there is a decrease in the Na^+^/K^+^-ATPase current and function. Increased [Na^+^]i levels are harmful in heart failure because they can lead to cardiac myocyte remodeling and systolic dysfunction. Furthermore, cardiac myocyte SGLTs are enhanced in T2DM, which also increases [Na^+^]i levels, resulting in arrhythmogenesis and oxidative stress of the myocardium of the diabetic patient [43]. Elevated [Na^+^]i levels also augment Ca^2+^ leakage in the sarcoplasmic reticulum, which increases the risk of arrhythmias and promotes myocardial dysfunction [110]. Since the ability to process cytoplasmic Ca^2+^ is reduced in heart failure, Ca^2+^ overload can block mitochondrial Ca^2+^ transporters, resulting in energy deficiency and oxidative stress, and ultimately cardiac remodeling and cell death [45]. SGLT-2 inhibitors can reduce [Na^+^]i levels by inhibiting the Na^+^/H^+^ exchange (NHE) system (primarily NHE1), reducing Ca^2+^ leakage, and enhancing mitochondrial Ca^2+^ concentration to improve heart function and improve outcomes in heart failure [111,112]. The measurement of cardiac biomarkers may provide important insights into the effects of these novel therapeutics.

### 6.1. Troponins

Troponins are structural and functional proteins present within the myocardium that comprise an essential element of the myocardial contractile unit [113]. Troponins I, T, and C constitute the structural elements of the troponin complex. Highly sensitive modern assays allow for the detection of troponins at low (nanogram or picogram) serum levels [114]. Troponins are released during injury to the myocardium, as in the case of myocardial ischemia or infarction, or pulmonary embolism [115]. Other causes may include myocardial apoptosis, neurohormonal excitotoxicity, and inflammatory processes [116]. Elevated troponins are associated with hospital mortality, increased length of hospital stay, and higher rehospitalization rates [117]. Troponin testing has increased the ability to detect acute coronary syndromes but faces limitations since troponin elevation may be associated with noncoronary heart diseases such as renal disease, sepsis, or rhamdomyolysis [118]. Furthermore, troponin levels exhibit diurnal and seasonal changes [119].

### 6.2. Troponin Levels in Heart Failure

Heart failure is associated with troponin elevation, and serum troponin levels are correlated with outcomes of heart failure [120]. In addition to its diagnostic value, troponins are used as prognostic indicators of adverse outcomes in heart failure as well [55]. Arenja et al. found that patients with acute heart failure had higher levels of cardiac troponin I on presentation than those with elevation from a non-cardiac etiology [120]. Furthermore, a three-year follow-up study by Demir et al. found that measurements of cardiac troponin T can independently predict long-term morbidity and mortality [121]. These findings can help identify patients with worsening heart failure at an earlier stage and guide their management [121]. In one meta-analysis of 9000 patients, troponin T was strongly associated with HF-related hospitalizations and cardiovascular mortality [122].

### 6.3. Troponins and SGLT-2 Inhibitors

Studies suggest that SGLT-2 inhibitors may reduce wall stress, induce the blockade of the sodium/hydrogen ion exchanger, and reduce cardiac necrosis, cardiac fibrosis, and ventricular loading via reduction in preload and afterload [77]. Such changes can result in a decrease in high-sensitivity cardiac troponin concentrations. High-sensitivity cardiac troponin assays remain superior at detecting myocardial necrosis compared to conventional assays [123].

In a study of prediabetic metabolic syndrome rats, empagliflozin showed improved cardiac hypertrophy and interstitial fibrosis following myocardial infarction [124,125]. A separate study of prediabetic rats with cardiac injury showed that the administration of dapagliflozin attenuated myocardial infarct size [125,126]). These cardiac structural improvements may result in a reduction in serum troponin concentrations.

In one prospective cohort study, 35 patients with T2DM were offered empagliflozin or incretin-based therapy (liraglutide or sitagliptin) in combination with insulin and metformin [127]. The blood viscosity and carotid artery wall shear stress significantly decreased in the empagliflozin group, while no change was detected in the control group. This reduction in wall stress may translate to a reduction in serum troponin concentrations. In another study of 97 patients with T2DM and CVD, empagliflozin reduced left ventricular mass [128]. This could be due to a decreased wall stress, which can result in reduced troponin levels. In a study of 49 patients with T2DM and coronary artery disease randomized to dapagliflozin or vildagliptin for 6 months, a reduction in systolic blood pressure and high-sensitivity troponin assay (hs-cTn) was observed in the dapagliflozin group but not in the vildagliptin group [129]. In another study, patients with T2DM who had or were at risk for atherosclerotic CVD were randomized to receive either dapagliflozin or a placebo [130]. Patients with higher concentrations of hs-cTn had higher rates of cardiovascular death or hospitalizations for heart failure. Furthermore, dapagliflozin consistently reduced the relative risk of heart failure hospitalizations or cardiovascular death, regardless of baseline hs-cTn. Although more data is needed, the results of these studies seem to imply that a reduction in hs-cTn concentrations may help explain some of the cardiovascular benefits observed with the use of SLGT-2 inhibitors, such as improved cardiac necrosis, cardiac fibrosis, and ventricular preload and afterload [77].

### 6.4. Natriuretic Peptides

Atrial natriuretic peptide (ANP) is produced by atrial and, to a lesser extent, ventricular myocardium and functions to reduce blood pressure and promote diuresis [131]. Synthesis is encoded by the *NPPA* gene on chromosome 1, and the active protein displays a half-life of approximately two minutes [132]. It is also synthesized in extracardiac tissues such as the kidneys [133]. Synthesis begins with the transcription of pre-proANP, followed by cleavage to proANP, which is the primary secreted form [134]. Upon secretion, proANP is cleaved to ANP and NT-proANP by the cardiac serine protease corin. Atrial stretch is the primary stimulus for ANP production as it often follows increases in circulating volume, elevated sodium load, and hypertension. Acute increases in atrial stretch induce the release of ANP, while chronic increases induce both release and synthesis [135]. Catecholamine release, RAAS activation, and endothelin may all act as additional triggers. [133]. ANPs are essential in preserving cardiac homeostasis and are used as a diagnostic and prognostic tool in heart failure patients [136].

**Figure 4:**
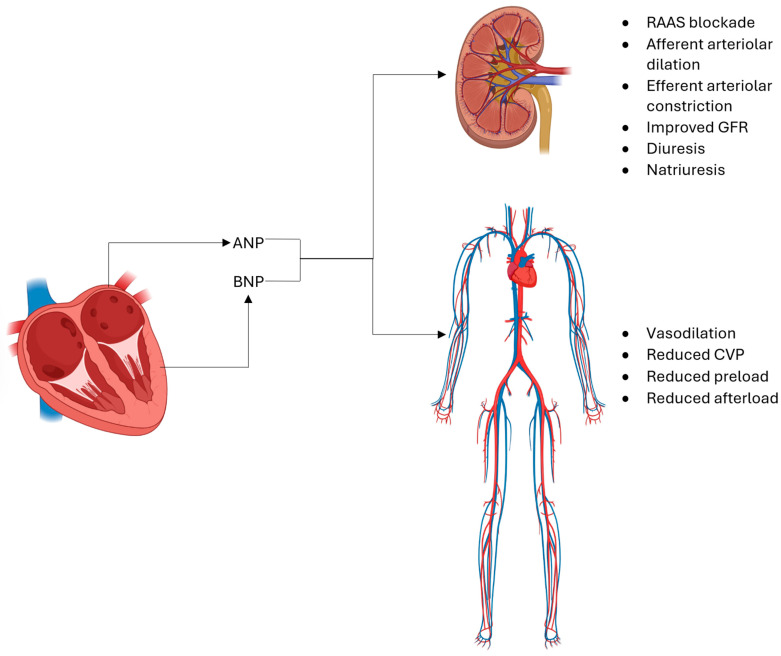
ANP and BNP act to improve renal blood flow, reduce total body water and sodium content, and reduce systemic vascular tone via multiple mechanisms, including natriuresis, diuresis, vasodilation, and offloading of systemic vascular pressures. ANP—atrial natriuretic peptide; BNP—brain natriuretic peptide.

ANP can also improve renal function by promoting renal perfusion via afferent arteriolar dilation and efferent arteriolar constriction, both of which work to improve glomerular filtration [137]. ANP blocks the reabsorption of sodium at the proximal tubule and the inner medullary ducts, thus improving urine production and natriuresis [138,139]. It also reduces blood pressure by blunting sympathetic signaling, increasing vascular permeability and venous capacitance [140]. These actions are mediated by the inhibition of aldosterone, RAAS, endothelin, and catecholamines [141]. (Figure 4) Another beneficial effect of ANP is that it can prevent cardiac hypertrophy [132]. The treatment of cultured cardiac myocytes with ANP inhibits angiotensin-II- and endothelin-1-mediated cell growth [142].

Brain-type natriuretic peptide (BNP) is a peptide neurohormone that is produced by cardiac ventricular myocytes in response to mechanical stretching due to excessive volume [133,143]. This process starts with the transcription of preproBNP, which is then cleaved to proBNP. proBNP is cleaved by the intracellular endopeptidase furin to the physiologically active C-terminal BNP and the inactive N-terminal fragment NT-proBNP [144]. Both are released in equimolar amounts, though with different half-lives (20 min for BNP and 120 min for NT-proBNP) [145]. While small amounts of BNP are stored in atrial granules, it is mainly produced and released from ventricles in direct response to myocardial stretching without intracellular storage [146].

Changes in the activity of corin and furin, and consequently ANP and BNP, have been linked to the progression and severity of HF [147]. Reduced corin activity contributes to the blunting of the normal ANP-mediated response to volume overload with resultant edema, systolic and diastolic dysfunction, and development of HFrEF [148]. In addition to the activation of BNP, furin acts on over 400 substrates, and thus may impact the development of HF via multiple mechanisms [149]. Elevated plasma levels of furin are associated with increases in blood pressure, lipid levels, incidence of T2DM, and major cardiovascular events [150,151,152,153].

### 6.5. Natriuretic Peptides and SGLT-2 Inhibitors

Most of the data available on SGLT-2 inhibitors and their effects on biomarkers are on the natriuretic peptides. The primary mechanism by which SGLT-2 inhibitors reduce plasma volume is through inducing glycosuria and osmotic diuresis [47]. In a study of 66 patients treated with empagliflozin, patients with and without T2DM had NT-proBNP concentrations that were unchanged at 4 weeks [154]. Similarly, in another study of 75 patients with T2DM randomized to placebo vs. dapagliflozin vs. hydrochlorothiazide, NTproBNP concentrations were unchanged at 12 weeks [155]. In a post hoc analysis of a randomized, double-blind, placebo-controlled study of canagliflozin, NT-proBNP concentrations were increased with placebo but were slightly decreased with canagliflozin over a 2-year study period [156].

The effects of SGLT-2 inhibitors have both a therapeutic and preventative effect on natriuretic peptide concentrations in patients with HF. In the DAPA-HF trial, dapagliflozin showed improved reduction in NT-proBNP concentration compared to placebo [157]. In another study by Nassif et al., of the patients with HFrEF randomized to dapagliflozin vs. placebo, no difference was found in the proportion of patients with a ≥20% decrease in NT-proBNP at 6 weeks, but at 12 weeks, a greater proportion of patients in the dapagliflozin group had a ≥20% decrease in NT-proBNP [158]. A recent study by Tanaka et al. demonstrated a slower rise in NT-proBNP in patients with T2DM and HFpEF receiving canagliflozin compared to placebo, though statistical significance was not met [159]. However, there is little data on the short-term effects of SGLT-2 inhibitors when used in patients with acute heart failure. In a study of 80 patients with acute HF with and without T2DM, empagliflozin had no effect on the NT-proBNP concentration levels but was still associated with favorable outcomes on combined endpoints of worsening heart failure, rehospitalization rates, and death at 60 days [160].

There is also data supporting the downregulation of ANP and BNP plasma levels with the use of SGLT-2 inhibitors. In a study by Feng et al., patients who were randomized to SLGT-2 inhibitors had lower ANP and BNP concentrations at 24 weeks compared to placebo control, with adjustment for baseline values [161]. In another study of 35 patients with T2DM and HFrEF who were randomized to empagliflozin vs. placebo, patients who received empagliflozin demonstrated significantly decreased ANP and BNP levels compared to baseline at all times [162]. On the other hand, the stratification of SGLT-2 inhibitor response by the severity of heart failure shows differences in changes to NT-proBNP levels [163]. Further data and studies are needed to clarify how natriuretic peptides can be used to predict cardiovascular risks in patients receiving SGLT-2 inhibitors.

### 6.6. Galectin-3

Galectin-3 (Gal-3) is a chimeric β-galactoside-binding protein within the lectin family that is responsible for a variety of intra- and intercellular processes such as apoptosis, differentiation, cell–cell adhesion, cell growth, and tissue repair. It is expressed in multiple extracellular, mitochondrial, nuclear, and cell surface environments [164,165]. Its variety of functions are attributed to its sites of expression. Gal-3 may be used prognostically and diagnostically as a biomarker for various disease processes, as it is quickly synthesized and released from inflamed or damaged cells [166]. Recent data also suggests that Gal-3 levels within serum and myocardium may be valuable as markers of inflammation and fibrosis in patients with HF [164]. Since ventricular remodeling is associated with fibrosis and inflammation of the myocardium, Gal-3 may contribute to the pathogenesis of heart disease [167]. The expression of Gal-3 is generally low; however, synthesis and secretion may increase with heart failure. The primary locations of Gal-3-binding sites in the myocardium are the matrix, fibroblasts, and macrophages. The secretion of Gal-3 into the extracellular space at sites of cell damage has been demonstrated, and it may serve an important function in the activation of fibroblasts [168].

In a case–control study by Khadeja Bi et al., plasma levels of Gal-3 plasma were 71% specific and 92% sensitive for the diagnosis of congestive heart failure at the threshold level of 8 ng/mL [168]. In an observational study by Huang et al., 223 heart failure patients were observed. They found that those patients had significantly greater serum Gal-3 concentrations than the control group [169]. Therefore, Gal-3 was proposed as a reliable indicator of heart failure with a sensitivity and specificity of 76.0% and 71.9%, respectively, at a threshold of 16 ng/mL [169,170]. In patients with ambulatory heart failure, increased Gal-3 levels were associated with increased mortality, suggesting that Gal-3 can be used as an indicator for long-term outcomes [171].

Though data are limited, Gal-3 levels at present seem minimally affected by the use of SGLT-2 inhibitors. In one post hoc analysis, the use of SGLT-2 inhibitors was associated with mildly increased serum Gal-3 levels over baseline; however, the difference was not statistically significant relative to placebo [156]. Further studies are necessary to delineate the relationship between SGLT-2 inhibitors and Gal-3.

### 6.7. Soluble Suppression of Tumorigenicity 2

The suppression of tumorigenicity 2 is part of the interleukin (IL)-1 receptor family and exists in two isoforms: membrane-bound (ST2L) and soluble (sST2) [172]. In addition to an intracellular signal initiation receptor domain, ST2L contains one transmembrane and three external IgG domains. sST2 lacks the intracellular and transmembrane domains [173,174,175]. sST2 production has been demonstrated within the myocardium and cardiac fibroblasts, and patients experiencing HF express elevated sST2 levels [176]. sST2 promotes myocardial hypertrophy, fibrosis, and apoptosis [177]. This eventually leads to irreversible damage after myocardial ischemia and infarction and to heart failure with poor prognosis [178,179]. IL-33 is released by cardiac stromal cells and extracardiac tissues in response to cellular activity [180]. IL-33 attaches to ST2L and reduces myocardial fibrosis and hypertrophy in response to angiotensin II or phenylephrine and induces antiapoptotic factors [179].

An sST2 concentration of greater than 35 ng/mL has been suggested as a reference value for the stratification of patients at high risk of heart failure [181]. The PRIDE trial demonstrated the value of sST2 in predicting 1-year mortality in patients with acute dyspnea secondary to destabilized HF [182]. The study proposes a threshold of sST2 ≥ 35 ng/mL as a predictor of poor prognosis and risk of death [183]. Compared to BNP, sST2 is minimally affected by renal clearance and may be used as an additional diagnostic marker for heart failure [184]. Both NTPro-BNP and sST2 show high diagnostic accuracy, but sST2 demonstrates greater predictive power for poor outcomes, including in-hospital mortality and mortality at one month [180]. Yamamoto et al. assessed the value of several biomarkers for risk stratification in patients with HF, including sST2, pentraxin 3, Gal-3, and hs-TnT [184]. sST2 levels were linked to poor outcomes such as hospitalization, cardiovascular mortality, and all-cause mortality in patients suffering from acute decompensated HF [184]. Therefore, the measurement of sST2 levels is a valuable tool for risk stratification, either by itself or in conjunction with troponins and natriuretic peptides.

Studies to date have shown little association between sST2 levels and SGLT-2 inhibitors. In an analysis of 666 patients treated with canagliflozin vs. placebo, sST2 levels were unchanged in both groups at 26, 52, and 104 weeks [156]. sST2 has been recently added to AHA guidelines to risk-stratify patients with HF [185].

### 6.8. Biomarkers of Inflammation

Inflammation is increasingly recognized as a key mechanism behind the development of CVD and HF, particularly in diabetic patients [186]. Of the range of soluble markers of inflammation, several have been correlated with changes in cardiovascular function and HF, including interleukin 6 (IL-6), c-reactive protein (CRP), and TNF-α [187]. Thus, some of the protective effects of SGLT-2 inhibitors in HF may be explained, at least in part, through anti-inflammatory mechanisms as evidenced by their effects on biomarkers of inflammation. This has been demonstrated in in vitro and animal studies and human clinical trials.

In a study by Mancini et al., the treatment of human endothelial cells with canagliflozin resulted in the reduced expression of IL-6 [96]. The use of various SGLT-2 inhibitors in multiple rodent models of T2DM has repeatedly demonstrated reductions in IL-6 levels [188]. Also, in a recent meta-analysis by Gohari et al., 5300 patients across 18 studies demonstrated a significant reduction in IL-6 levels when treated with SGLT-2 inhibitors, either as monotherapy or as part of a multi-agent regimen [189].

Mouse and rat models of treatment with SGLT-2 inhibitors have shown reductions in CRP [188]. In a systematic review by Bray et al., 10 out of 12 included clinical studies demonstrated reductions in CRP following SGLT-2 inhibitor treatment [190]. Similarly, a meta-analysis by Sun et al. of data from 927 patients showed significant decreases in CRP levels in patients treated with SGLT-2 inhibitors [191].

An anti-inflammatory assay of mouse macrophages following treatment with canagliflozin has demonstrated the reduced secretion of TNF-α [192]. This finding was consistent with results from in vivo mouse studies using a variety of SGLT-2 inhibitors [188]. In a systematic review by Bray et al., two out of four studies demonstrated TNF-α reductions following SGLT-2 inhibitor treatment [190]. These findings were reproduced in a separate randomized controlled trial comparing empagliflozin to glimepiride [193].

The expression of monocyte chemoattractant protein-1 (MCP-1) in kidney proximal tubule cells has been shown to be reduced following exposure to empagliflozin [194]. Similar findings have been reported in mouse adipose tissue [195]. The treatment of diabetic mice with SGLT-2 inhibitors showed significant MCP-1 reduction, though there is a substantial difference in effect size across various agents [188]. In one observational study, SGLT-2 inhibitor therapy was correlated with significantly lower MCP-1 levels than other diabetic medications [196]. Also, in a subgroup analysis of the CANVAS trial, patients treated with canagliflozin versus placebo showed reductions in urinary MCP-1 levels [197].

## 7. Clinical Implications

Biomarker testing allows for risk prediction and therapeutic monitoring in patients with CVD and HF. A retrospective analysis of 1000 patients from the SMART study showed that multiple biomarker testing is predictive of major adverse cardiovascular events in T2DM [198]. However, much remains unknown about the variability of biomarker responses between individuals and across various types and severities of HF. One meta-analysis of natriuretic peptide testing in HF showed significant differences in the cutoff values used across various studies [199]. Such differences make the interpretation of results and their applicability to clinical practice and future trials difficult.

Additionally, the relation between individual SGLT-2 inhibitors, biomarker changes, and clinical outcomes shows significant variability. While DAPA-HF showed reductions in both NT-proBNP and adverse HF outcomes in patients treated with dapagliflozin, the DEFINE-HF trial showed improvement in HF symptoms but not in NT-proBNP levels [158,200]. In an observational study by Nuzzi et al., NT-proBNP levels decreased in patients with non-advanced HF treated with SGLT-2 inhibitors, while levels increased in patients with advanced HF [163]. Additionally, in in vitro studies by Mancini et al., anti-inflammatory effects on human endothelial cells were demonstrated by canagliflozin but not by dapagliflozin or empagliflozin [96].

The complex interaction between SGLT-2 inhibitors, cardiac biomarkers, and HF necessitates more than simple assumptions or algorithms in the application of their use. Future directions will rely heavily on more sophisticated technologies such as machine learning, neural networks, and iterative algorithms to clarify these relationships and personalize treatment approaches for individuals. Multiple artificial intelligence models have already shown promise in predicting adverse outcomes and treatment response based on individual biomarker profiles [201].

## 8. Conclusions

This review highlights the relationship between SGLT-2 inhibitors and various biomarkers of HF. As the use of SLGLT-2 inhibitors is projected to increase significantly in the coming years, the need to understand the complex ways in which these drug–biomarker interactions occur and affect symptoms becomes increasingly pressing [202]. We have discussed several well-investigated biomarkers, their role in the pathogenesis of HF, and how SGLT-2 inhibitors interact at a cellular and clinical level. Despite encouraging evidence, further human trials are needed to better clarify the extent and nature of the connection between SGLT-2 inhibitor use and changes in biomarkers of HF.

## Abbreviations

The following abbreviations are used in this manuscript:

AHA	American Heart Association
ANP	Atrial natriuretic peptide
ATP	Adenosine triphosphate
BNP	B-type natriuretic peptide
CRP	C-reactive protein
CVD	Cardiovascular disease
Gal-3	Galectin-3
HF	Heart failure
HFmrEF	HF with mildly reduced ejection fraction
HFpEF	HF with preserved ejection fraction
HFrEF	HF with reduced ejection fraction
hs-cTn	High-sensitivity troponin assay
IL	Interleukin
NT-proBNP	N-terminal pro-B-type natriuretic peptide
RAAS	Renin–angiotensin–aldosterone system
ROS	Reactive oxygen species
SGLT-2	Sodium–glucose cotransporter 2
sST2	Soluble suppression of tumorigenicity-2
TNF-α	Tumor necrosis factor alpha
T2DM	Type 2 diabetes mellitus
